# Boosting SARS-CoV-2 immunity in immunocompromised individuals

**DOI:** 10.1038/s41435-023-00219-6

**Published:** 2023-12-19

**Authors:** Thomas R. Müller, Marcus Buggert

**Affiliations:** Department of Medicine Huddinge, Center for Infectious Medicine, Karolinska Institutet, Stockholm, Sweden

**Keywords:** Immunological memory, Infection, Viral infection

## Background

Since its inception, SARS-CoV-2 has continued to impose unprecedented challenges on the medical and scientific communities. The complex and ever-evolving nature of the virus, particularly through the emergence of multiple variants of concern (VOCs) has substantially intensified these challenges. Emerging VOCs not only possess the potential to spread more efficiently but can also possibly evade the human immune response, thus affecting the efficacy of currently available vaccines.

In the battle against the SARS-CoV-2 virus, one group has continuously faced heightened risks – individuals with compromised immune systems, such as patients on immunosuppressive medications or patients with underlying health and genetic conditions. These individuals frequently respond insufficiently to COVID-19 vaccines, which puts this population at a higher risk of severe COVID-19 outcomes and necessitates improved strategies to enhance their protective immunity [1, 2].

## Findings

Our recent investigation in *Science Translational Medicine* offers promising evidence for such a strategy: the administration of booster vaccine doses in immunocompromised individuals [3]. While numerous studies have examined the role of antibodies post-vaccination, the ability of Omicron to evade this arm of adaptive immunity has shifted our attention toward another crucial component – T cells. The immune cells are essential for a well-rounded and effective response to viral infections For instance, in the case of breakthrough SARS-CoV-2 infections, there is a rapid and substantial resurgence of memory T cell populations. This resurgence strongly correlates with accelerated viral clearance, providing evidence of their significant role in inhibiting viral replication during breakthrough infections [4].

Through our research, we observed that booster vaccinations led to a substantial increase in both CD4^+^ and CD8^+^ T cells against the spike protein in immunocompromised individuals and the elderly. Despite reduced neutralizing antibody responses to the Omicron variant, our study revealed that T cell responses are significantly increased against Omicron after booster vaccination, particularly in individuals who responded poorly to their initial vaccine doses. Importantly, within our large cohort, no individuals developed severe disease during the Omicron wave, even though 22% were infected. This suggests that booster vaccinations could compensate for inherent weaker immunity, potentially offering substantial protection against severe COVID-19.

Uncovering the quantitative enhancement of T cell responses following booster vaccinations in immunocompromised individuals paints just half the picture. The other half, equally pivotal, lies in deciphering the functional quality of the immune response. Our data revealed a distinct shift toward a more pronounced CD8^+^ T cell response following booster vaccination. Among CD8^+^ T cells, we found that many spike-specific cells displayed a ‘T effector memory RA (TEMRA)’ phenotype but maintained attributes of stemness. These TEMRA cells were capable of self-renewal and differentiation, and notably, they expressed higher levels of TCF-1, indicating enhanced proliferation and functional capacity. The stem-like characteristic of these TEMRA cells, signifying their long-term persistence in the body and readiness to mount a robust cytotoxic response upon virus re-encounter, provides a potent, ready-for-action defense against SARS-CoV-2. The heightened readiness, facilitated by booster doses, could significantly reduce the risk of severe outcomes upon infection, including with emerging variants of concern.

Tackling SARS-CoV-2, particularly in the face of new VOCs, requires not just a potent immune response, but one that is diverse and adaptable. The need for a varied arsenal led us to investigate the role of ‘hybrid immunity’ in augmenting T cells against COVID-19. Our paper has revealed how hybrid immunity enhances and diversifies the T cell response to SARS-CoV-2. We found that vaccinated individuals, upon subsequent infection with the Omicron variant, showed formation of new T cell clones, thus broadening the overall repertoire of SARS-CoV-2-reactive T cells and enhancing the body’s adaptable defenses.

Lastly, we noticed an enhanced response to both spike and non-spike epitopes in individuals previously infected with Omicron. While current vaccines mainly target the spike protein of the virus, our findings suggest that other structural and non-structural proteins can also trigger responses. The diversified T cell response following an Omicron infection demonstrates the flexibility and adaptability of T cells based on an enormously large combinatorial pool of T cell receptors and SARS-CoV-2 peptides presented by a highly diverse set of human leukocyte antigens (HLA) throughout the human population. Hence, hybrid immunity creates a comprehensive, multi-target defense against the virus, bolstering the body’s capacity to combat future encounters with SARS-CoV-2.

## The need for further research

While our findings offer promising insights into the robust and diverse T cell responses elicited by booster vaccinations and Omicron infection, it is important to highlight the limitations and implications for future research. A complex aspect of studying T cell responses lies in the immense diversity of epitope-HLA combinations, with each individual having a unique repertoire of memory T cell clones. To fully understand the nuanced shifts in this repertoire post-Omicron infection, larger sample sizes and higher-resolution data are necessary. Furthermore, developing more effective ways to map epitopes would provide a more detailed picture of how the T cell repertoire evolves in response to variant infections. The ongoing evolution of SARS-CoV-2, with the emergence of new VOCs, further underscores the need for continuous monitoring and research to keep pace with the ever-changing landscape of the virus and its impact on the immune system.

## Conclusions

Our study highlights the important role of booster vaccinations in securing an effective immune response against SARS-CoV-2, particularly among immunocompromised individuals. The importance of booster vaccinations is emphasized by their ability to enhance T cell responses, particularly of cytotoxic CD8^+^ T cells, and promote the formation of stem-like TEMRA cells. These alterations in the immune landscape not only confer protection against the current strains but may also have implications for the body’s defenses against future variants. Our observations on hybrid immunity provide a nuanced understanding of how a previous encounter with the virus, in combination with vaccination, can help diversify the body’s defenses to provide a broader range of viral targets.

These findings strongly advocate for the continuation and expansion of large-scale vaccination campaigns, inclusive of booster doses. They also stress the importance of continued research into the complex and dynamic interplay between the evolving VOCs and our immune systems. The future of combating this global pandemic depends on our ability to adapt strategies in line with our growing understanding of this virus and immune responses to it in vulnerable people.
